# Effects of 6 Weeks of Traditional Resistance Training or High Intensity Interval Resistance Training on Body Composition, Aerobic Power and Strength in Healthy Young Subjects: A Randomized Parallel Trial

**DOI:** 10.3390/ijerph17114093

**Published:** 2020-06-08

**Authors:** Tatiana Moro, Giuseppe Marcolin, Antonino Bianco, Francesco Bolzetta, Linda Berton, Giuseppe Sergi, Antonio Paoli

**Affiliations:** 1 Department of Biomedical Sciences, University of Padova, 35122 Padova, Italy; giuseppe.marcolin@unipd.it (G.M.); antonio.paoli@unipd.it (A.P.); 2 Department of Psychology, Educational Science and Human Movement, University of Palermo, 90133 Palermo, Italy; antonino.bianco@unipa.it; 3 Department of Medicine, University of Padova, 35122 Padova, Italy; francesco.bolzetta@aulss3.veneto.it (F.B.); linda_berton@libero.it (L.B.); giuseppe.sergi@unipd.it (G.S.); 4 Department of Medicine, Geriatric Unit, Azienda ULSS3 “Serenissima”, 30031, Dolo, 30035 Mirano District, Italy

**Keywords:** resistance training, high intensity, recovery time, physical fitness, strength

## Abstract

Consistent practice of physical activity has well known positive effects on general health; however, time for exercise remains one major barrier for many. An acute bout of high intensity interval resistance training (HIIRT) increases acute resting energy expenditure (REE) and decreases respiratory ratio (RR), suggesting its potential role on weight loss and increased fatty acid oxidation. The aim of this study was to test the long-term effect of HIIRT on body composition, lipid profile and muscle strength using a randomized parallel trial. Twenty healthy young adults (22.15 ± 1.95 years) were randomized to perform either a HIIRT (N = 11) protocol, consisting of three sets of 6 repetitions at 6 repetition maximum (RM) and then 20 seconds of rest between repetitions until exhaustion repeated for 3 times with 2′30″ rest between sets or a traditional training (TRT, N = 9) protocol of 3 sets of 15 reps with 75 sec of rest between sets. Body composition, resting energy metabolism, aerobic capacity, muscle strength and blood measurements were taken before and after 8 weeks of training. Both protocols enhanced muscle strength, but only HIIRT improved endurance strength performance (+22.07%, *p* < 0.05) and lean body mass (+2.82%, *p* < 0.05). REE and RR were unaltered as lipid profile. HIIRT represents a valid training method to improve muscle strength and mass, but its role on body weight control was not confirmed.

## 1. Introduction

Resistance training (RT) is an important component of exercise protocols, mostly used to enhance muscle strength and hypertrophy. Additionally, RT can influence resting energy expenditure (REE) and fat metabolism, assisting in weight loss process [1].

Lack of time is one of the major barriers to a constant practice of physical activity, therefore the concept of “high intensity interval training” (HIIT) is becoming very popular. This kind of training is principally used with aerobic exercise and allows to achieve maximal results in a relatively short time of effort. In the past ten years, HIIT has been largely investigated and different protocols have been defined, but all of them are characterized by brief repeated bouts of vigorous exercise alternated with periods of lower intensity exercise or recovery [2,3,4]. Because RT is composed by numerous variables (i.e., sets, repetition, load, type of contraction) [5], it is difficult to replicate this same pattern of short-term high intensity training. We have previously identified a variant of the rest-pause [6] or so-called “cluster training” technique [7], named high-intensity interval resistance training (HIIRT) [8], as a good candidate to mimic HIIT and reduce the total training time [9,10]. This technique requires to lift heavy loads with a partial recovery within a single set, promoting, on one hand, total depletion of intramuscular creatine phosphate [11] and complete exhaustion [12] after each set with a partial recovery [6], and on the other, a shorter total time to finish the exercise.

We have already demonstrated that an acute bout of HIIRT increases excess post-exercise oxygen consumption (EPOC) and REE and shifts the respiratory quotient (RR) towards fatty acids utilization [13]. These findings suggested that HIIRT has a potential influence on weight loss and body composition. Some research speculates that hormonal mechanisms related to muscle hypertrophy and muscle damage are involved in EPOC increase, through alteration of cell homeostasis [13,14]. EPOC represents the increased consumption of oxygen (VO_2_) used to repay the oxygen debt contracted in the initial phase of effort [15]. From a physiological point of view, muscle tissue is one of the main limiting factors for VO_2_ due to the important role of its oxidative enzymes, mitochondria and capillary perfusion on aerobic capacity. However, the role of high intensity RT on aerobic capacity is still controversial, with some authors showing no changes in aerobic capacity [16,17] and others confirming its role on empowering energy metabolism [17,18,19].

It is well established that physical activity improves lipid profile and reduces cardiovascular risk [20]; in particular, RT plays an important role on reducing total cholesterol, LDL-cholesterol, TG and increased HDL-c [21]; however, the effects of high intensity RT have not been completely explicated. Recently, we demonstrated that HIIRT improves lipid profile in healthy and obese older adults [10,22]; however, no data are available on younger adults.

Thus, the purpose of this research was to evaluate the long-term effects of HIIRT compared to a traditional resistance training (TRT) program on resting metabolism, body composition and some blood values in sedentary young people; we also wanted to verify the effects on aerobic capacity and different expressions of strength. Compared to TRT, HIIRT is expected to offer a good training stimulus but with a shorter exercise time commitment. Based on our previous findings [9,10], our hypothesis was that HIIRT would have a greater effect on implement muscle strength and reduce fat mass compared to a traditional training protocol (TRT). In addition, because of the acute influence of HIIRT on EPOC, we expected a reduction of respiratory quotient and an increase in aerobic capacity.

## 2. Materials and Methods

### 2.1. Subjects

Twenty-one young healthy subjects (22.15 ± 1.95 years, BMI 23.57 ± 1.63 kg/m^2^) were recruited from the University of Padova student community via advertisements in social media and websites. However, one participant was for final analysis (CONSORT diagram is presented in Appendix A). excluded during the final analysis due to poor compliance with exercise protocol. Thus, twenty subjects were considered.

All subjects were recreationally active and had previous experience with resistance training, but none of them practiced regularly strength training (<2 sessions/week). Eligibility was determined using clinical history and physical exam. Exclusion criteria for the study included history of chronic diseases (diabetes, cardiorespiratory or metabolic disorders) or any other condition that might have interfered with one’s ability to adhere to exercise protocols. Baseline subjects’ characteristics are reported in Table 1.

### 2.2. Study Design

The study was designed as a randomized parallel trial. After signing a written informed consent form, eligible subjects were randomly assigned to either a high intensity interval resistance training (HIIRT, N = 11) or a traditional resistance training (TRT, N = 9) protocol. The randomization list was generated using GraphPad QuickCalcs Web site (http://www.graphpad.com/quickcalcs).

**Figure 1:**
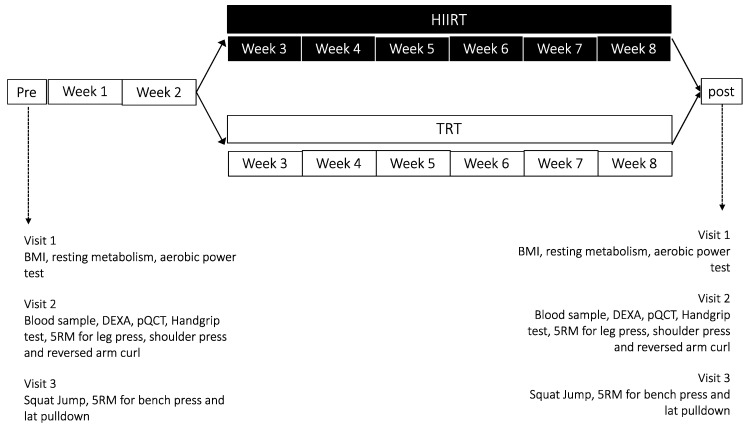
Study design. HIIRT, High Intensity Interval Training; TRT, Traditional Resistance Training; DEXA, Dual-Energy X-Ray Absorptiometry; pQCT, Peripheral Quantitative Computed Tomography; RM, Repetition Maximum.

Subjects came to our lab three times for screening tests and to familiarize themselves with the training protocol. The general design is depicted in Figure 1. The first visit was used to obtain the subject’s BMI, resting metabolism through REE and RR measurement and aerobic power (VO_2max_) via an incremental maximal test at the cycle-ergometer. Three days later, we obtained blood samples and body composition analysis, whilst muscle strength was assessed with a handgrip and a 5-repetition maximum (RM) test for lower and upper limb. During the third visit, 5RM test was performed to assess chest and back strength, and a squat jump test was used to test muscle power performance.

After the completion of all tests, subjects started their training protocol. All subjects underwent the same TRT training schedule for the first two weeks, and from weeks 3 to 8, they performed the assigned interventional protocol (HIIRT or TRT) three times per week. After the conclusion of the above, subjects were asked to return to the laboratory, where they repeated all tests in the same order as at the pre-training stage. Subjects were also asked to fill a food diary the week before starting and ending the study.

The study was approved by the Human Ethical Commission of the Department of Biomedical Sciences (HEC-DSB 01/2015), in accordance with Helsinki’s declaration of 1995 as modified in 2000. All participants started the experimental procedures in February 2013, and study was concluded in May 2013.

### 2.3. Body Composition Assessment

Body mass index (BMI) was calculated in kg/m^2^: Body weight was measured using an electronic scale to the nearest 0.01 kg (Tanita BWB-800 Medical Scales, USA) and height using a portable stadiometer with a precision of 0.01 m (Holtain Ltd., UK).

Total and regional lean mass and adipose tissue were analyzed by dual-energy x-ray absorptiometry (DEXA) (QDR 4500 W, Hologic Italia s.r.l, Rome, Italy) after an overnight fast.

Muscle mass percentage and its ratio with lean body mass were also calculated using a skin-fold method. The formula to estimate body composition considered nine skin folds sites (triceps, biceps, chest, subscapular, iliac, abdominal, anterior and popliteal thigh), 6 bone circumferences (arm, forearm, waist, hips, thigh and calf) and 4 bone diameters (elbow, wrist, knee and ankle). Skinfolds were measured to the nearest 1 mm using a Holtain caliper (Holtain Ltd., UK). All measurements were taken by the same operator (AP) before and after the study, according to standard operating procedures [23]. Data obtained were then processed on a validated software (Fitnext^®^, Caldogno, Vicenza, Italy) [24].

Distal thigh and forearm muscle section were also analyzed using a Norland/Stratec XCT-3000 Peripheral Quantitative Computed Tomography (pQCT) scanner (Stratec Medizintechnik GmbH, Pforzheim, Germany), which is a non-invasive technique normally employed to measure bone density but also useful for highlighting the transverse section of muscle belly (CSA) in mm^2^. Subjects were placed on a special stool and asked to remain as still as possible while the scanner moved around the limbs throughout their length. The obtained images were then processed using analysis software (XCT 3000-Stratec Medizintechnik GmbH, Tumeltsham, Austria) to quantify muscle and adipose tissue content. As per standard procedures, all measurements were taken and measured by the same operators (FB and LB).

### 2.4. Muscle Strength and Aerobic Performance

Strength tests were divided into four groups: Dynamic (1RM), two isometric handgrip strength tests (peak and endurance) and explosive strength (squat jump test).

During the 6RM test, subjects were asked to reach the load with which they were able to perform a maximum of 6 repetitions. This method is particularly safe when the participant has no experience with training [25]. Maximal repetition was estimated using Brzycki’s formula [26]. All muscle groups employed during training session were tested in alternate days.

Isometric force was measured with the Dynatronic 100 ergometer handgrip (New Mechanics Pastorelli, Gallarate, VA, Italy), and the average of 3 performances was considered. To evaluate endurance strength, subjects were asked to maintain pressure on the dynamometer at 50% of their ceiling for the longest possible time. Time was recorded and considered as indicative of muscle endurance capacity.

Finally, a squat jump test was performed according to Bosco description [27] and the best out of three performances was chosen for analysis. Subjects were asked to keep hands on their hips, and the trunk was maintained erect. Subjects started from the seated position, with 90° of knee flexion, on a contact mat (Ergojump—Bosco system, srl, S. Rufina di Cittaducale, Rieti, Italia). The height of jumps was calculated according to the Asmussen and Bonde–Petersen formula [28].

Aerobic power was measured with an incremental maximal test on a ciclo-ergometer. Peak power output (PPO), VO_2_ (L/min), VO_2_ max (mL/kg/min), VCO_2_ (L/min), carbon dioxide end-tidal partial pressure (PETCO_2_), pulmonary ventilation (VE) and RR were measured. Respiratory gases were measured via standard open-circuit calorimetry (max Encore 29 System, Vmax, Viasys Healthcare, Inc., Yorba Linda, CA, USA) on a breath-by-breath modality. The protocol included 3 min of warm up at 60 W, then 1 min at 100 W and intensity increased by 15 W every 60 s until exhaustion. Subjects were asked to keep a pedaling cycle of 60 rpm for the duration of the test; the test was interrupted by the subject due to exhaustion, or when the pedaling cycle was not maintained.

### 2.5. Resting Metabolism

Resting energy expenditure (REE) and Oxygen uptake (VO_2_) were measured using the above-mentioned calorimetry system. Participants arrived after an overnight fast and rested in a comfortable supine position before beginning the test. Then, a silicone mask covered both mouth and nose was applied, and subjects were asked to stay relaxed but awake during the test. Oxygen and carbon dioxide concentrations were obtained for a total of 20 min. Only the last 10 min were used to measure the results and to calculate resting energy expenditure (REE) and respiratory ratio (RR) using the modified Weir equation [29].

### 2.6. Blood Biochemistry

Blood samples were obtained approximately at 8 A.M. at rest, and after an overnight fast, subjects were instructed to avoid any strenuous activity during the 24 h prior to the test.

Total cholesterol (CHOLt), high-density lipoprotein cholesterol (HDL-C), low-density lipoprotein cholesterol (LDL-C), glucose, uric acid, creatinine and gamma-glutamyl transferase (GGT) were measured by an enzymatic colorimetric method using a Modular D2400 (Roche Diagnostics, Basel, Switzerland).

Insulin was measured with a chemiluminescent immunoassay (Siemens Immulite 2000); alanine aminotransferase (ALT) and aspartate transaminase (AST) were measured by pyridoxal phosphate activation according to manufactured instruction, whilst a kinetic enzymatic method was used to detect creatine kinase (CK) and Urea.

Total testosterone was determined by the immunochemiluminescent method (Roche Cobas e601, Roche Diagnostics, Mannheim, Germany), whilst free testosterone was measured with a radioimmunological manual method (Beckman Coulter). Insulin-like growth factor-1 (IGF-I) was measured using the analyzer Liaison XL (DiaSorin S.p.A, Vercelli- Italy), whilst its isoform IGFBP1 with a sandwich immunoassay based on a chemiluminescent revelation (Dia Source).

### 2.7. Training Protocol

All subjects trained three times per week with at least one-day rest between sessions. All participants began their training with two weeks of TRT familiarize with the exercise protocol. Training load for each exercise was assessed based on the initial 5RM test and adapted in relation to the improvement that occurred with training. As such, during the first two weeks, all subjects performed the protocol with an intensity of 15RM (corresponding at ~60% of 1RM).

TRT consisted of 3 sets of 15 repetitions at 60% 1RM with 1′15” rest between sets. The training session lasted approximately 62 min, including the warm-up period.

HIIRT technique consisted of a first set of 6 repetitions at 80% 1RM (corresponding to 6RM) followed by a 20” rest; then, subjects were asked to lift the same weight until failure (habitually 2 or 3 repetitions) followed by another 20” rest period with repetitions to fatigue. This sequence counted as one set; then, the subjects rested 2′30” before performing a second set (6 + 2 + 2 reps). The training session took approximately 43 min including the warm-up period.

The exercise protocol was kept for the entire time of the study and was identical for both groups. Training program consisted in a total body workout, and the following sequence of exercises was included leg press for lower limb, bench press for chest muscles, lat pulldown for back muscles, military press for shoulders and reverse arm curl for biceps. All subjects were asked to perform each set to exhaustion and to increase the exercise load according.

Because all subjects had previous experience with resistance exercise, training sessions were not directly supervised; however, subjects were asked to complete a workout log and send it to the research team after each session.

### 2.8. Statistical Analysis

The analysis was performed via Prism 8.0 GraphPad software (Abacus Concepts GraphPad Software, San Diego, USA). Sample size was obtained assuming an interaction of a Root Mean Square Standardized Effect (RMSSE) of 0.25 with fixed power of 80% and an alpha risk of 5% for the main variable (muscle strength measured as 1RM). Normality was confirmed via the Shapiro–Wilk’s W test. After assessing that there were no baseline differences between groups through an independent samples t-test, an ordinary two-way ANOVA for repeated-measures was performed (using time as the within-subject factor and training as the between-subject factor) in order to assess differences between groups over the course of the study. Post-hoc analyses were performed using the Bonferroni test. Pearson correlation was used to assess the correlation between body composition techniques. Differences were considered significant at *p* < 0.05. Data are presented as mean ± standard deviation.

## 3. Results

All subjects completed the study indicating a full retention with both protocols. However, one subject failed to report exhaustively compliance with the training program and was excluded from the final analysis.

### 3.1. Body Composition

**Figure 2:**
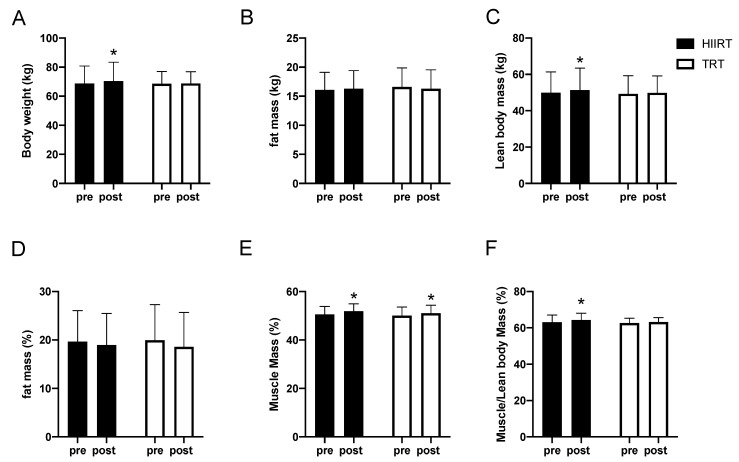
Body composition analysis. * Significantly different from baseline (*p* < 0.05). HIIRT, High Intensity Interval Training; TRT, Traditional Resistance Training.

After 8 weeks of training, a significant time x treatment interaction (*p* = 0.04) was detected for total body mass, with an increase in body mass in HIIRT (+2.27%, *p* = 0.004) but not in TRT group (+0.36%, *p* > 0.05) (Figure 2A). Similarly, lean body mass measured by DEXA significantly increased in HIIRT (+2.82%; *p* = 0.0004) but not in TRT (+1.29%; *p* > 0.05) (Figure 2C). A main time effect (*p* = 0.03) was detected in fat mass percentage, which decreased in both groups without significant differences between treatments (Figure 2D). Similar results were detected when body composition was estimated via skinfolds. Using this methodology, we were also able to distinguish muscle mass from lean body mass. The percentage of muscle mass significantly increased in both groups (HIIRT + 2.66, *p* = 0.001; TRT + 2.02%, *p* = 0.024); however, muscle to lean body mass ratio was higher only in HIIRT group (+1.18%, *p* = 0.04) compared to TRT (+0.96%, *p* > 0.05) as represent d in Figure 2E,F.

We also performed a correlation between body composition results assessed by DEXA and skinfold algorithm to ensure compatibility between the two techniques. As presented in Appendix B, fat mass (r^2^ = 0.89, *p* < 0.0001), lean mass (r^2^ = 0.97, *p* < 0.0001) and percentage of body fat (r^2^ = 0.89, *p* < 0.0001), were highly correlated.

### 3.2. Muscle Area

Table 2 presents pQCT results on the distal thigh and forearm cross-sectional analysis. We observed a significant increase in forearm total area in both groups (HIIRT + 4.22, *p* < 0.0001; TRT + 3.16, *p* = 0.004), which matched with an increase in muscle mass (HIIRT + 4.38%; TRT + 7.73%) and a decrease in fat area (HIIRT − 2.46%; TRT − 5.40%). Any significant difference was found in the distal thigh area, except for the fat area, which significantly decreased only in TRT (−8.56%, *p* = 0.01). Although not significant, we observed a general decreased in total (HIIRT − 3.78%, TRT − 6.67%), and thigh muscle area (HIIRT − 3.48%; TRT − 6.56%).

### 3.3. Muscle Strength

Isometric strength measured via handgrip did not improve significantly (HIIRT + 4.06%, TRT + 0.54%, *p* > 0.05). However, the endurance test revealed a significant improvement in the HIIRT group (+22.07%, *p* = 0.02) compared to TRT (+12.27%, *p* > 0.05).

Squat jump performance improved in both groups (HIIRT + 26.02%, TRT + 14.89%) without any significant difference between groups or from baseline.

All 1RM tests presented a significant main effect of time (*p* < 0.05), and both groups improved their dynamic strength in all the exercises analyzed. However, no differences were detected between protocols. Strength results are presented in Table 3.

### 3.4. Basal Metabolism and Ventilatory Parameters

As showed in Table 4 we did not observe any significant difference in basal metabolism. Respiratory ratio decreased by 0.90% in HIIRT group and by 1.29% in TRT group. REE decreased by 4.66% and 1.69%, respectively, in HIIRT and TRT; when normalized by lean body mass, the differences were more pronounced in HIIRT (–8.33%) compared to TRT (–3.57%) but without statistical significance.

We observed a general improvement in the incremental test performed at cyclo-ergometer in both groups; however, any of the parameters analyzed were significantly different from baseline. PPO increased in HIIRT by 9.89% (*p* = 0.06) and by 7.33% in TRT (*p* > 0.05); when normalized by body weight, the differences were similar (HIIRT + 7.55%, TRT + 7.03). VO_2max_ increased by 16.23% in HIIRT compared to 20.50% in TRT, and similarly, when expressed for body mass, HIIRT improved by 11.15% compared to 18.97% in TRT. Pulmonary ventilation was 20.36% higher in HIIRT and 11.33% in TRT; however, the difference was not significant from baseline.

### 3.5. Blood Parameters

As described in Table 5, any relevant difference was observed in any of the blood parameters analyzed.

## 4. Discussion

One of the main goals of training programs is to improve muscle performance, and at the same time, try to implement cardiovascular fitness and optimize body composition. We have previously demonstrated that an acute bout of a modified rest-pause technique, which we have named HIIRT, increased resting energy expenditure and enhanced fat oxidation [9]. Based on these findings, we advanced the hypotheses that HIIRT may improve body composition and resting metabolism as well as enhancing muscle strength and aerobic capacity compared to a traditional training method (TRT). The main finding of this study was that long-term HIIRT protocol improved muscle mass and strength; however, its role on body weight control and blood lipid reduction was not confirmed in healthy young adults.

The beneficial effect of exercise on many health parameters is granted when training is practiced regularly. Is thus necessary to optimize training intervention to elicit the positive effect of physical activity. In young adults, it seems that exercise intensity and time to complete the session [30] are the two main factors that influence training adherence. In the present study, we considered two different modalities of resistance training, which differed in duration and intensity. In both cases, we observed a full adherence to training protocols, indicating that HIIRT is tolerated as much as a traditional resistance training program. It is worth to note that the subjects enrolled in the study were young and highly motivated; we have indeed observed that in older adults engaged in longer period of training (6 months), the retention in HIIRT was higher compared to a lower intensity and longer time commitment training [10].

During RT programs, muscle strength and performance can be enhanced by accurate management of their different components such as sets, repetitions or rest periods [5,31]. Manipulating inter-repetition rest time—in particular, reducing intervals between repetitions during a set—allows both to increase the chance of achieving muscular failure and reduce the total time necessary to complete the same volume of training. HIIRT technique is built upon this concept; indeed, our participants completed their training session ~20 min earlier compared to TRT group, which corresponded to approximately a 30% reduction in total training time.

After 8 weeks, HIIRT group presented a significant rise in body weight due to an increase in lean body mass compared to TRT. These results are quite interesting, considering that the position stand of the American College of Sport Medicine suggests that a higher volume of training (1–3 sets of 8–12 reps at 70–85% 1RM) should be employed with novice individuals to maximize muscle hypertrophy [32]. Training volume (number of sets and repetition) seems to be a crucial factor to promote muscle gains and is normally associated with lower loads. Greater volume increases time under tension, which is known to be an essential driver of muscle hypertrophy [33,34] but requires higher repetition to achieve muscular fatigue. Volitional fatigue is another important component to promote muscle anabolism [35], which seems to guarantee similar hypertrophic results even at lower loads (30–60% 1RM) [36]. On the other hand, heavier loads stimulate markers of hypertrophy (i.e., myoglobin and LDH) [37,38] and force production [39,40] but require a higher number of sets to promote peripheral fatigue. However, when higher loads are employed, complete muscular exhaustion seems to be not strictly necessary [41] for muscle hypertrophy. HIIRT technique breaks a regular set of 10-12 reps in smaller “cluster” sets with lower repetitions and short-set intervals, which translated into a greater volume of training achieved with a particular high load (>80% 1RM). This allows to obtain both higher volume and intensity, most likely increasing the hypertrophic response to mechanical stress also in the novel trainee [39,42].

The importance of muscular failure is still a controversial topic also in regard to its role in maximizing muscle performance [43,44]. With the short-term interval between sets, HIIRT induces a total depletion of intramuscular creatine phosphate and complete exhaustion after each set. In addition, some studies showed that similar cluster training is able to preserve movement speed and power output during each set, reducing neuromuscular fatigue [45]. We observed that HIIRT and TRT had the same effect on muscular strength, confirming that higher loads are not the “condictio sine qua non” to obtain an effectiveness strength response to RT. The effect of training load, in fact, seems to be connected to the former training experience [32], but this is not the case of our participants, which were novice to RT. It is plausible to believe that the greater load entity used with HIIRT may have ensured complete recruitment of motor units, a condition necessary to improve both strength and muscle hypertrophy [46]. These would also explain the response obtained during the handgrip tests: We did not observe any difference on maximum strength expression, whilst HIIRT group achieved significantly greater improvements on the endurance test compared to TRT group. The short resting time between sets does not allow complete recovery by the glycolytic fibers (normally involved in high intensity exercise) and therefore requires the additional intervention of slow fibers in order to conclude the set. This condition, repeated over time, has probably induced a better muscle adaptation to local fatigue compared to TRT, during which the motor unit’s activation is less intense and, therefore, less stimulating in increasing resistance to muscle exhaustion.

Based on our previous research [9,10,47], we expected a reduction of fat mass and higher resting metabolism after HIIRT protocol compared to a more traditional training method. We indeed observed an increase in the post exercise REE and a reduction in the RR towards oxidation of fats in response to an acute bout of HIIRT [9]. Lower fasting RR is associated with a reduced risk of obesity and cardiovascular disease [48,49] and thus has important implications for general health. In the present study, we were not able to replicate the shift of RR, which decreased only by up to 1% with HIIRT; moreover, REE decreased by up to 5–6% in both groups. Although some authors believe that a reduction of REE is a possible consequence of intense training [50,51], this cannot explain the countertrend observed in the current study. Post-exercise REE increases within 30 h from the end of the session [52] but not beyond this period; in the study published in 2012 [9], we measured the effect of HIIRT 22 h after the training session, and that could have reflected the prolonged increase in energy cost due to the training intensity, which is not being translated into a chronic adaptation as demonstrated in the present study. It is possible that, for non-experienced subjects, the intensity of HIIRT was such as to implement compensatory mechanisms to reduce resting energy expenditure. Some studies conducted on elite athletes have proved that they responded to intense workouts with an increase in energy intake, while sedentary and unaccustomed subjects tend to reduce energy expenditure [51]. In support of the latter hypothesis, subjects randomized in HIIRT group reported an increased feeling of hunger compared to TRT group, which instead managed to keep their dietary regime unchanged.

Both types of training improved maximal aerobic power but without obvious differences between groups. Controversy continued to exist as to which RT modality is the best to maximize oxygen consumption. Some authors seem to prefer protocols with high loads, few repetitions and long recoveries, but there are just as many studies that instead seem to indicate opposite directions. Ozaki [53] analyzed the literature regarding the effect of different variables of RT on aerobic power and concluded that, in younger adults, training volume or intensity seems not to have a significant role on VO2max improvement. However, it appears that short rest periods between sets (<30 sec) may enhance oxygen consumption due to greater cardiovascular demand. Moreover, the majority of the studies showed an increase in the work rate and/or test duration, without a concomitant improvement in VO_2max_ [54,55]. We observed similar effects on VO_2max_, work rate and test duration, confirming that muscle strength and anaerobic capacity are necessary to improve maximum aerobic power [56], since these components are fundamental to guarantee access to muscle energy resources.

Finally, in regard to the lipid profile and other blood parameters, we did not observe any effect of training or differences between the two groups. These results can be justified by the excellent state of health of our participants. Indeed, we have already demonstrated that in older adults, HIIRT was able to reduce basal insulin levels, total cholesterol and LDL, suggesting a positive effect on insulin sensitivity and parameters associated with cardiovascular risk [10].

Some limitations of the present study should be taken into account. One is that dietary intake was uncontrolled. Subjects were asked to maintain their habitual caloric intake, as measured during the preliminary week of the study. However, it is possible that differences in energy or nutrient intake during the study and between groups could have existed and played a role in the slight increase in fat mass observed in HIIRT group. We already mentioned that participants assigned to HIIRT referred an increased hunger during the training period, and this may have translated into greater caloric intake. Secondly, although subjects were asked to fill a training report for each session, training was not regularly supervised. This may have affected the achievement of the proper intensity during each session. Moreover, the training volume between protocols was not perfectly equal, and that may have influenced the muscular response in terms of hypertrophy and strength. Thus, future research should normalize the training load in order to assess more precisely the effect of this particular cluster technique on muscle performance and body composition.

## 5. Conclusions

In summary, the results of the present study suggest that non-expert young adults can safely perform high intensity interval resistance training (HIIRT). HIIRT increases muscle mass and muscle strength in a shorter training session. However, HIIRT did not improve body fat percentage or resting energy expenditure as expected, suggesting that this training modality is not enough to promote alone fat metabolism.

Lack of time is one of the principal obstacles to the regular practice of physical activity, thus a protocol that allows to obtain proper levels of muscle mass and performance and simultaneously guarantees a high adherence rate is essential to promote a healthy life style. HIIRT seems to be a good training alternative for those who do not have much time to exercise.

## Figures and Tables

**Table 1:** Subjects characteristics at baseline.

	HIIRT (*N* = 11)	TRT (*N* = 9)
Age (years)	22.27 ± 1.85	22.00 ± 2.18
Height (cm)	171.36 ± 10.34	170.78 ± 9.76
Weight (kg)	68.76 ± 12.06	68.60 ± 8.39
BMI (kg/m^2^)	23.67 ± 2.07	23.45 ± 0.97
Fat mass (%)	23.94 ± 5.64	24.74 ± 6.69

Data are mean ± SD. HIIRT, High Intensity Interval Training; TRT, Traditional Resistance Training; BMI, Body Mass Index.

**Table 2:** Muscle area measurements via peripheral quantitative computed tomography (pQCT) scan.

	HIIRT (*N* = 11)		TRT (*N* = 9)	
	Pre	Post	Pre	Post
Forearm total area (mm^3^)	5081.45 ± 919.94	5299.05 ± 982.13 *	4903.00 ± 747.16	5052.94 ± 732.91 *
Forearm muscle area (mm^3^)	3869.66 ± 1239.69	4016.45 ± 1192.34	3532.00 ± 824.47	3805.81 ± 949.33
Forearm fat area (mm^3^)	842.39 ± 469.04	905.43 ± 391.67	1032.39 ± 396.77	900.31 ± 343.13
Forearm muscle/fat ratio (%)	26.80 ± 19.22	26.56 ± 16.92	33.08 ± 17.91	26.94 ± 15.79
Thigh total area (mm^3^)	16,438.36 ± 3277.39	15,782.18 ± 3431.63	16,126.17 ± 15,555.64	15,011.67 ± 1689.93
Thigh muscle area (mm^3^)	10,523.73 ± 3200.85	9911.64 ± 2638.34	10,084.89 ± 2165.65	9287.36 ± 2715.81
Thigh fat area (mm^3^)	5130.30 ± 2214.18	5033.32 ± 2434.48	5355.78 ± 1700.94	4986.89 ± 1648.49 *
Thigh muscle/fat ratio (%)	56.31 ± 37.36	55.37 ± 32.07	57.34 ± 26.83	62.76 ± 38.54

Data are mean ± SD. * significantly different from pre (*p* < 0.05). HIIRT, High Intensity Interval Training; TRT, Traditional Resistance Training.

**Table 3:** Muscle strength.

	HIIRT (*N* = 11)		TRT (*N* = 9)	
	Pre	Post	Pre	Post
Handgrip (*N*)	38.63 ± 10.90	40.28 ± 12.55	39.91 ± 9.28	40.50 ± 11.87
Handgrip Endurance strength (sec)	82.14 ± 45.64	94.41 ± 45.59 *	72.63 ± 20.65	81.50 ± 26.65
Squat Jump (sec)	0.32 ± 0.10	0.36 ± 0.08	0.29 ± 0.12	0.35 ± 0.08
Leg press (kg)	157.47 ± 33.29	207.37 ± 45.36 *	167.93 ± 33.60	199.54 ± 29.67 *
Bench press (kg)	55.20 ± 23.87	66.60 ± 22.41 *	67.17 ± 18.81	59.25 ± 18.20 *
Lat pulldown (kg)	64.20 ± 22.16	79.58 ± 22.42 *	59.25 ± 18.20	73.01 ± 19.57 *
Shoulder press (kg)	14.65 ± 4.95	23.05 ± 7.60 *	17.14 ± 7.60	21.96 ± 7.83 *
Reverse arm curl (kg)	22.02 ± 7.23	31.74 ± 9.31 *	21.75 ± 6.28	27.01 ± 5.62 *

Data are mean ± SD. * significantly different from pre (*p* < 0.05). HIIRT, High Intensity Interval Training; TRT, Traditional Resistance Training; *N*, Newton; sec, seconds.

**Table 4:** Basal metabolism and ventilatory parameters during the incremental test.

	HIIRT (*N* = 11)		TRT (*N* = 9)	
	Pre	Post	Pre	Post
**Basal Metabolism**				
Respiratory Ratio	0.82 ± 0.07	0.81 ± 0.05	0.82 ± 0.05	0.82 ± 0.04
REE (kcal/die)	1832.12 ± 392.21	1733.62 ± 422.31	1681.22 ± 437.76	1571.16 ± 397.68
REE/lean mass (kcal/die/kg)	0.04 ± 0.00	0.03 ± 0.01	0.03 ± 0.01	0.03 ± 0.01
**Incremental test**				
PPO (watt)	196.06 ± 52.40	213.01 ± 53.39	195.37 ± 50.97	209.33 ± 56.37
PPO/BW (watt/kg)	2.85 ± 0.54	3.02 ± 0.47	2.82 ± 0.49	3.02 ± 0.60
VO_2_ (L/min)	2.81 ± 1.07	3.09 ± 0.80	2.55 ± 0.81	2.92 ± 0.86
VO_2_/kg (ml/kg/min)	41.42 ± 11.29	44.47 ± 9.13	37.59 ± 9.44	42.47 ± 8.88
VCO_2_ (L/min)	3.00 ± 1.11	3.32 ± 0.94	2.73 ± 0.81	3.09 ± 0.93
Respiratory Ratio	1.07 ± 0.07	1.07 ± 0.05	1.08 ± 0.06	1.06 ± 0.06
VE (L/min)	81.58 ± 27.92	93.78 ± 26.61	85.37 ± 30.23	91.53 ± 33.46
PetCO_2_ (mmHg)	41.22 ± 5.12	39.67 ± 4.51	37.49 ± 5.37	39.00 ± 5.22
Test duration (sec)	528.09 ± 232.20	578.09 ± 232.20	538.22 ± 204.95	556.11 ± 229.57

Data are mean ± SD. HIIRT, High Intensity Interval Training; TRT, Traditional Resistance Training; REE, Resting Energy Expenditure; PPO, Peak Power Output; BW, Body Weight; VO_2_, Oxygen Consumption; VCO_2_, Carbon dioxide output; VE, pulmonary ventilation; PETCO_2_, carbon dioxide end-tidal partial pressure.

**Table 5:** Blood parameters.

	HIIRT (*N* = 11)		TRT (*N* = 9)	
	Pre	Post	Pre	Post
CHOL (mmol/L)	4.07 ± 0.64	4.09 ± 0.64	4.51 ± 0.78	4.32 ± 0.57
HDL (mmol/L)	1.37 ± 0.38	1.35 ± 0.40	1.29 ± 0.33	1.37 ± 0.31
LDL (mmol/L)	2.15 ± 0.54	2.17 ± 0.50	2.61 ± 0.70	2.51 ± 0.65
Glucose (mmol/L)	4.92 ± 0.34	4.93 ± 0.30	4.84 ± 0.31	4.66 ± 0.32
ALT (U/L)	10.82 ± 5.12	11.36 ± 3.64	9.38 ± 2.88	10.56 ± 4.19
AST (U/L)	26.45 ± 5.84	26.00 ± 6.00	24.13 ± 3.44	25.33 ± 2.55
Gamma GT (U/L)	12.36 ± 4.84	11.09 ± 3.11	10.38 ± 4.21	10.11 ± 4.08
Creatine kinase (umol/L)	125.45 ± 110.18	94.36 ± 45.88	100.50 ± 57.68	135.33 ± 99.45
Creatinine (umol/L)	86.09 ± 12.76	83.73 ± 13.15	87.25 ± 13.84	84.56 ± 13.44
Uric acid (mg/L)	0.28 ± 0.04	0.28 ± 0.03	0.27 ± 0.08	0.27 ± 0.06
Urea (mg/L)	5.32 ± 1.57	5.35 ± 1.52	5.58 ± 1.39	5.07 ± 0.95
**hormones**				
Cortisol (nmol/L)	460.55 ± 113.99	409.27 ± 123.86	489.95 ± 126.26	437.89 ± 126.72
IGF-1 (ng/mL)	293.73 ± 66.41	309.85 ± 75.53	348.36 ± 69.38	340.68 ± 83.05
IGF-BP1 (ng/mL)	4.15 ± 2.21	3.47 ± 2.68	2.68 ± 2.32	2.59 ± 1.61
Free Testosterone (ng/mL)				
Male	60.85 ± 9.36	55.22 ± 7.95	55.31 ± 17.79	60.84 ± 12.56
Female	5.12 ± 0.50	4.92 ± 0.66	4.27 ± 1.96	4.80 ± 2.30
Total Testosterone (ng/mL)				
Male	22.64 ± 5.22	20.29 ± 4.20	20.59 ± 4.79	23.02 ± 2.60
Female	1.59 ± 0.23	1.50 ± 0.38	1.46 ± 0.89	1.39 ± 0.83

Data are mean ± SD. HIIRT, High Intensity Interval Training; TRT, Traditional Resistance Training; CHOL, total cholesterol; HDL, High-density lipoprotein cholesterol; LDL, Low-density lipoprotein cholesterol; ALT, alanine Aminotransferase; AST, Aspartate Aminotrandferase; Gamma GT, Gamma-glutamyl Transpeptidase; IGF-1, Insulin-like Growth Factor-1; IGF-BP1, Insulin-Like Growth Factor-Binding Protein 1.
